# Unexpected differences between planar and column liquid chromatographic retention of 1-acenaphthenol enantiomers controlled by supramolecular interactions involving β-cyclodextrin at subambient temperatures

**DOI:** 10.1007/s00216-017-0313-y

**Published:** 2017-03-24

**Authors:** Hatsuichi Ohta, Elżbieta Włodarczyk, Krzysztof Piaskowski, Aleksandra Kaleniecka, Lucyna Lewandowska, Michał J. Baran, Mariusz Wojnicz, Kiyokatsu Jinno, Yoshihiro Saito, Paweł K. Zarzycki

**Affiliations:** 1 Department of Environmental and Life Sciences, Toyohashi University of Technology, 1-1 Hibarigaoka, Tempakucho, Toyohashi, 441-8580 Japan; 2 Department of Environmental Technologies and Bioanalytics, Faculty of Civil Engineering, Environmental and Geodetic Sciences, Koszalin University of Technology, Sniadeckich 2, 75-453 Koszalin, Poland

**Keywords:** β-Cyclodextrin, 1-Acenaphthenol enantiomers, Micro-planar chromatography, High-performance liquid chromatography, Supramolecular interaction, Temperature effects

## Abstract

We report the results of experimental work focusing on host-guest supramolecular complex creation between macrocyclic compound (β-cyclodextrin) and 1-acenaphthenol enantiomers (racemic mixture) in liquid phase composed of 35% acetonitrile in water (*v*/*v*) at different temperatures ranging from 0 to 90 °C. Experimental setup involved several analytical protocols based on classical non-forced flow planar chromatography (RP-18 TLC plates), micro-TLC (RP-18 W HPTLC plates), column chromatography (HPLC with C-18 and C-30 stationary phases), as well as UV-Vis spectrophotometry and optical microscopy. It has been found that under various planar chromatographic conditions (stationary plates type, chamber shape and volume, development mode, and saturation) non-typical retention properties (extremely high retention) of 1-acenaphthenol at subambient temperatures can be observed. To our knowledge, reported experimental results are in opposition to currently described retention models based on column chromatographic investigation of host-guest complexes (where in case of strong interaction of given analyte with macrocyclic mobile phases additive, which itself is non strongly retarded by stationary phase—close to the retention of dead volume marker, the retention of target compounds is shortened at low temperatures). To explain this TLC phenomenon that may have in our opinion a number of practical applications, especially for selective high throughput separation involving microchromatographic and/or microfluidic devices as well fractionation and extraction protocols (using, e.g., bar extraction systems), several experiments were conducted focusing on (i) acenaphthenol chromatography under different instrumental conditions, (ii) cyclodextrin retention measured as analyte or mobile phase additive, (iii) plate development time under different mobile phases and temperature settings, (iv) various column chromatographic conditions including C-30 and two C-18 stationary phases, (v) UV-Vis spectrophotometry, and (vi) microscopy inspection of precipitated CD-acenaphthenol crystals. Analysis of collected data has revealed that the most probable reasons for TLC retention behavior of 1-acenaphthenol under β-cyclodextrin additive conditions can be associated with (i) solubility changes of created host-guest complex, (ii) kinetics of solid complex precipitation, and (iii) differences in analysis time between planar and column chromatography. Because precipitation phenomenon may have a massive impact on analytes quantification involving macrocycles as the mobile phase additives, our previously reported data concerning a number of low-molecular compounds (mainly steroids and non steroidal endocrine disrupting chemicals) using HPLC methodology based on binary mobile phases without and with β-cyclodextrin and its hydroxypropyl derivative were re-examined and results discussed. Considering these data and the whole data set reported presently, the enhanced model of chromatographic retention driven by host-guest interaction was proposed.

**Electronic supplementary material:**

The online version of this article (doi:10.1007/s00216-017-0313-y) contains supplementary material, which is available to authorized users.

## Introduction

Cyclic oligosaccharides form a wide group of low-molecular mass donut-like-shaped compounds. An important class of such molecules are cyclodextrins (CDs), particularly native β-cyclodextrin and its derivatives [1, 2]. Fairly unique physicochemical properties of cyclodextrins result from their chiral three-dimensional shape and particularly, outside of the donut surface location of polar hydroxyl groups [3–7].

Within congeneric sequence of native cyclodextrins (α, β, and γ), the β-CD is one magnitude less soluble in water compared to its α- and γ-CD neighbors [8]. This property may limit given applications of β-cyclodextrin for inclusion systems in pharmacy, analytical chemistry, and technological processes, where high concentration of host molecules is demanded or high solubility of the resulting complex is necessary, e.g., in liquid chromatography or for drug delivery systems [9, 10]. Some of the β-cyclodextrin derivatives, especially hydroxypropyl-β-CD, have virtually unlimited solubility in water. However, this is associated with strong increase of viscosity, particularly for macrocycles concentration more than 50% (*w*/*w*) [11]. Derivatization of native CDs rings or their immobilization on solid matrices within polymers net may significantly change the unique inclusion properties, which are specific for the native forms. Therefore, extensive research is still conducted for tuning of inclusion specificity for given target molecules [12, 13].

In spite of discovery and extensive studies devoted to other simple (crown ethers, calixarenes, cyclophanes, macrocyclic antibiotics) or more sophisticated, based on mechanically interlocked molecular architecture of macrocyclic compounds (like rotaxanes, catenanes, knotanes), there is still increasing interest in cyclodextrin research [14, 15]. This is mainly because of a number of key applications of CDs and extensive usage of macrocyclic oligosaccharides in chemistry (analytical chemistry, separation sciences; e.g., as resolution enhancers, active components of extraction systems) [16–18], micro-electronics and microfluidics devices (components of sensing systems) [19, 20], medicine, pharmacy, and cosmetology (drug and bioactive components carriers) [21–24], as well as food industry (aroma carriers, biocomponents extraction, active components of smart food packaging systems) [25–27]. Most recently, there is also an extensive research focusing on their efficient encapsulation properties, working as free molecules or after incorporation within new polysaccharide-based nanomaterials [28, 29]. It has been demonstrated that these highly selective absorption systems may reveal outstanding removal capabilities for certain micropollutants, particularly, for large-scale removing systems that are selective to endocrine-disrupting compounds (EDCs) or antibacterial drugs and low-molecular mass biocompounds [30–35]. The abovementioned micropollutants are very difficult to remove from given matrices (e.g., wastewater) using classical technologies involving commonly applied sorbents or commercial-activated carbons.

Due to the three-dimensional shape of cyclodextrins, they efficiently recognize low molecular mass stereoisomers and chiral compounds, which was extensively used in high-throughput separation systems (GC, HPLC, HPTLC), particularly that CDs are chemically stable under a wide range of pH conditions [9, 16, 36, 37]. Under planar chromatographic conditions, retention profiles of cyclodextrins on octadecylsilane (C-18) are strongly non-linear. Interestingly, elution of native CDs can be observed, even if there is practically no solubility of these macrocycles in given mobile phase, e.g., in pure methanol. In contrary, water cannot elute native cyclodextrins from C-18 stationary phases but CDs are very well water soluble, especially α- and γ-CD [38, 39].

Macrocyclic oligosaccharides-based complexes have been also recognized as the temperature supersensitive objects, acting under both static and dynamic conditions [40]. Temperature effects on chromatographic retention involving mobile phases modified with cyclodextrins (under given chromatographic conditions, in which CD additive is strongly eluted by binary organic-water mobile phase, for example acetonitrile:water 35:65 (*v*/*v*), allow to use the temperature as an effective way to control selectivity in liquid chromatography, similarly to gas chromatography, but using very narrow temperature range [41]. General phenomenological model describing such liquid chromatography retention controlled by supramolecular complexes creation is based on the observation that retention of inclusion complexes can be varied between two lines formed by the Van’t Hoff plot of free cyclodextrin and the Van’t Hoff plot of the uncomplexed solute [15, 42–49].

The main goal of the present experimental work focuses on reassessment of basic phenomenological model describing liquid chromatography retention controlled by supramolecular complexes creation, involving mobile phase additives, which are not strongly retarded by stationary phase. We demonstrated that for given molecules (1-acenaphthenol enantiomers), there is an enormous difference between column and planar chromatographic retention. To our knowledge, such behavior was not described in the literature before. To explain this phenomenon, several experiments were conducted focusing on (i) acenaphthenol chromatography under different instrumental conditions, (ii) cyclodextrin retention measured as analyte or mobile phase additive, (iii) plate development time under different mobile phases and temperature conditions, (iv) various column chromatographic conditions including C-30 and two C-18 stationary phases, (v) UV-Vis spectrophotometry, and (vi) microscopy inspection of precipitated β-CD-acenaphthenol crystals. Presented experimental data may have in our opinion a number of practical applications, especially for optimization of high throughput separation involving microchromatographic and/or microfluidic devices as well highly selective fractionation, extraction, and/or purification protocols using cyclodextrins as active molecules.

## Experimental

### Reagents and materials

1-Acenaphthenol (racemic mixture) was obtained from Aldrich-Chemie (Steinheim, Germany). β-Cyclodextrin (für biochemische Zwecke), acetonitrile, and methanol (LiChrosolv; HPLC grade) were products of Merck (Darmstadt Germany). Dead time marker (sodium nitrate; NaNO_3_) was purchased from POCh (Gliwice, Poland). Sulphuric acid (95% p.a.) was obtained from Chempur (Piekary Śląskie, Poland). Binary mobile phases were prepared using double-distilled tap water.

### Chromatography

Chromatographic standards were prepared in methanol (1 mg mL^−1^; acenaphthenol stock solution), water (for NaNO_3_), or 35% (*v*/*v*) acetonitrile in water mixture (for β-cyclodextrin). For HPLC experiments, stock solutions were diluted with mobile phase (acetonitrile:water, 35:65, *v*/*v*) to obtain injection solutions at concentration of 50 μg mL^−1^ (10 μg mL^−1^ for NaNO_3_). In case of planar chromatography, 1 μL volume of stock solution was manually transferred to plates starting line (1 μg/spot). Stock solution for planar chromatographic studies of β-cyclodextrin was prepared as 10 mM solution of macrocycle in acetonitrile:water 35:65 (*v*/*v*). From this stock solution, appropriate dilutions at concentrations of 1 and 5 mM were prepared and 1 μL of each was transferred to plate start line (β-CD quantities of 1.2, 5.8, and 11.6 μg/spot were chromatographed). HPLC, TLC, and HPTLC mobile phases were composed of acetonitrile:water mixtures (35:65, *v*/*v*) without or with addition of β-cyclodextrin at a concentration of 10 mM.

Column chromatographic separations were carried out using two HPLC systems consisting of:

(i) A HPLC pump (PU-980; JASCO, Tokyo, Japan) and a UV diode-array detector (MD-915 multiwavelength detector; JASCO, Tokyo, Japan). The components of interest were injected through a 20-μL loop using a Rheodyne 7125 injector (Cotati, CA, USA). The column temperature was controlled by a Thermo-Mate BF-61 (Yamato Scientific, Tokyo, Japan). HPLC system was operated with two 15 cm length (4.6 mm i.d.) analytical columns: Develosil C18-UG-5 (5 μm, 17.6 C%) and Develosil C30-UG-5 (18.2 C%) obtained from Nomura Chemical (Seto, Japan). Mobile phase flow rate was set at 0.5 mL min^−1^.
(ii) A HPLC pump (LC-10ADvp), injector (Rheodyne 7725i, Rohner Park, CA, USA) with 20 μL loop, a SPD-M20A photodiode array detection (DAD) system, and a computer system for data acquisition with software LC Solution (version 1.21 SP1; 2002–2005) manufactured by Shimadzu (Suzhou New District, Jiangsu, China). Mobile phase flow rate was set at 1.0 mL min^−1^. Column temperature was controlled by foam insulated water jacket connected to circulating thermostat (Nestlab RTE7; product of Thermo Electron Corporation, Newington, NH, USA). Temperature of DAD detector cell of this system was controlled independently and set at 30 °C. This system was operating with a Supelcosil LC-18 analytical column (10 cm × 4.6-mm internal diameter and 5-μm particle size) that was obtained from Supelco (Bellefonte, PA, USA).

Planar chromatographic separations (TLC, HPTLC) were conducted using two chambers and developing modes:

(i) Classical vertical chamber: 14 × 6 × 6 cm glass container (wall thickness 2 mm), which was placed in temperature-controlled water bath. Within this chamber, TLC plate was positioned vertically in saturated chamber and chromatography was carried out using TLC Silica gel 60 RP-18 F_254_S glass-based plates obtained from Merck (Darmstadt, Germany). Original plates (25 × 25 cm) were manually cut to working size 2 × 10 cm (developing distance 50 mm). TLC plate images were acquired using Apple iPhone 5s 8-megapixel iSight built-in digital camera.
(ii) Horizontal micro chamber unit described previously [50]. Briefly, 5 × 5 cm microplate with analytes spotted at the start line was horizontally positioned within removable unit, which was transferred to the temperature-controlled oven. A foam-insulated metal oven was connected to an external liquid circulating thermostat (Ultra-Low Refrigerated Circulator FP51-SL, Julabo, Seelbach, Germany) operating with ethanol as a circulating fluid. After 15 min of temperature equilibration, mobile phase (0.5 mL, approximately) was injected and chromatographic process was conducted within unsaturated chamber until mobile phase front reached the opposite edge of microplate (developing distance 45 mm). Chromatographic experiments involving this chamber were based on glass HPTLC RP-18 WF_254_S plates purchased from Merck (Darmstadt, Germany).

### TLC detection and data acquisition

Acenaphthenol spots were visualized using sulphuric acid:water 1:3 (*v*/*v*) mixture. After plate development, the mobile phase was dried at room temperature for 30 min, and plate was immersed in visualization liquid for 1 s. Then, wet plate was immediately heated in air circulating oven for 15 min at temperature of 70 °C.

β-cyclodextrin spots or as mobile phase additive were detected by plate exposure to iodine vapors for 5 min, after mobile phase drying at temperature of 80 °C for 10 min.

Spot patterns on microplates were acquired using Canon EOS1100D digital camera equipped with Tamron 55-200 lens (covered by HAMA filter UV 390/52 mm) from a distance of 94.5 cm under visible (F16; 1/4 s), UV 254 nm (F16; 8 s), and UV 365 nm (F16; 16 s) light conditions. Acquisition system was equipped with ring of 12 LED lamps (JDR, SMDHLCW-250; 3.5 W; 6400 K; 250 lm, Sanico Electronics, Warszawa, Poland) and two linear UV 365/254 nm light sources: VL-6.LC obtained from Vilber Lourmat (Cedex, France).

Quantitative retention data (spots position) were extracted from unprocessed digital images using ImageJ software (ver. 1.48 Wayne Rasband, National Institutes of Health, USA; http://rsb.info.nih.gov/ij). For micro-chromatograms images presented in the paper, a global manual balance filter was applied to increase the contrast for spots visual evaluation.

### UV-Vis spectrophotometry, optical microscopy, and light scattering

UV-Vis absorption spectra were recorded using Hewlett Packard one beam spectrophotometer (HP-8453, Fed. Rep. of Germany). All measurements were carried out using standard 1-cm-thick quartz cell under temperature-controlled conditions maintained by homemade anti-frosting thermostatic module described previously [51]. Temperature of this device was controlled by external circulating thermostat (Nestlab RTE7; product of Thermo Electron Corporation, Newington, NH, USA).

Light scattering from supramolecular crystals was induced by visible light laser beam (green light 532 ± 10 nm, <10 mW, 532 nm) passing through glass vial containing acenaphthenol at concentration of 10 μg mL^−1^ and β-cyclodextrin (10 mM) in acetonitrile:water water liquid phase, after 3 days at room temperature (22 ± 1 °C).

Optical microscope pictures of acenaphthenol/β-cyclodextrin crystals were acquired under visible light conditions using Motic BA310 LED microscop (Motic China Group, Xianmen, China) equipped with Moticam 3 MP USB CMOS digital camera, with the help of Motic Image Plus 2.0 software.

## Results and discussion

In gas (GC) or liquid (LC including HPLC and TLC) chromatography, Van’t Hoff plot may be used to model the response of target components retention to temperature. This fundamental relationship, presented as Eq. 1, is usually linear, and therefore, the changes of enthalpy and entropy can be estimated from the slope and intercept of given line:

1 $$ \ln k=-{\varDelta H}^o/\mathrm{R} T+{\varDelta S}^o+ \ln \varPhi $$

where *R* is gas constant, *k* denotes retention factor, ln*Φ* column phase ratio, *ΔH* ^*o*^ enthalpy change, and *ΔS* ^*o*^ entropy change of transfer of the analyte from the mobile phase to stationary phase. In practice, linear Van’t Hoff behavior was very well documented for a number of low-molecular mass analytes (e.g., *n*-alkanes, polycyclic aromatic hydrocarbons, or steroids) under various chromatographic conditions (normal, reversed phase, isocratic, or gradient elution systems) [46–49, 52–54].

According to experimental data available in literature, linear relationship between retention and inversed temperature can be expected if retention mechanism is the same over the temperature range investigated and considering that changes of enthalpy and entropy as well as phase ratio are temperature independent [47, 48]. However, for several liquid chromatographic systems, non-linear Van’t Hoff plots have also been reported, including separation of enantiomeric solutes, which are of great interest for biological and biochemical investigations [55–57]. As the main reasons for observed deviations from Van’t Hoff plots, the reversible processes altering absorption enthalpy or entropy have been highlighted. They include changes in analytes conformation and/or presence of multiple types of retention mechanisms, various types of binding sites, as well as phases transitions concerning chromatographic mobile or stationary phases [55, 57–59]. Under such conditions, the effect of temperature on liquid chromatographic retention might be complex and difficult to predict without experimental data for given temperature range. Noteworthy, reported nonlinearities can be effectively used for improvement of chromatographic separation (e.g., fullerenes via changes in structure of stationary phase) [60–62] and particularly, chiral separation (for example: D-(±)norgestrel or 1-acenaphthenol via changes in complexation strength occurring in mobile phase) [9, 63].

In liquid chromatographic systems, cyclodextrins as the host components of supramolecular complexes act in relatively complex environment, consisting of a number of competitive molecules: inorganic/organic additives of mobile phases and surface ligands of stationary phases. Therefore, prediction of target molecules retention is complicated, particularly at different temperatures since such complexes have been found as temperature supersensitive objects [64]. Competitive interaction with cyclodextrins is especially significant for molecules containing long *n*-alkanes chains. This was previously demonstrated involving both retention of cyclodextrins as analytes on C-18 stationary phases as well as spectrophotometry-based experiments for testing of complexation strength of various analytes with CDs [15, 38, 51, 64–67].

Most complete phenomenological model of reversed-phase chromatographic retention using cyclodextrins as the mobile phase additive, which was invented and extensively investigated by Sybilska, is based on the assumption that separation, including enantioseparation, can be affected by two phenomena: (i) adsorption of inclusion complexes on stationary phase and (ii) complexation of solutes in the bulk mobile phase solution [68]. It should be noted that in this system, cyclodextrins may work in two modes: strongly or weakly adsorbed on *n*-alkanes stationary phase. In practice, retention behavior of mobile phase macrocyclic additive can be precisely adjusted by concentration of organic solvent of binary liquids (e.g., by applying acetonitrile in water mixtures close to 35%; *v*/*v*), which can be easily visualized by planar chromatography experiments [38, 39, 64]. We presented a number of experimental data indicating that reversed-phase chromatographic systems, where cyclodextrin additive is weakly retarded by stationary phase (close to the retention volume of dead volume marker), are extremely temperature sensitive, and allow massive retention and selectivity changes for given low-molecular mass compounds [41]. Moreover, we documented that retention of inclusion complexes based on cyclodextrins as the host molecules can be varied between two lines formed by the Van’t Hoff plot of free cyclodextrin and the Van’t Hoff plot of the uncomplexed solute [15, 42, 43].

Previously, we investigated the ability of reversed-phase HPLC system with octadecylsilane (C-18) column for fast and high throughput separation of steroids stereoisomers and enantioseparation of 1-acenaphthenol involving native cyclodextrins and their hydroxypropyl derivatives [9]. Presented experimental data have revealed that eluent composed of acetonitrile:water (35:65, *v*/*v*) and modified with native β- and γ-cyclodextrins has the ability to separate optical isomers of 1-acenaphthenol. It has been observed that at subambient temperature (0 °C), the peak of the mixture of enantiomers was broadened (if γ-CD was applied). Baseline separation of acenaphthenol enantiomers (*R*_s_ = 1.62) was observed by applying β-CD as mobile phase additive at concentration of 10 mM and using 15 cm long C-18 column. Circular dichroism spectra revealed that under such conditions, the (−) acenaphthenol enantiomer is eluted first. Noteworthy, these data have indicated that chiral separation of 1-acenaphthenol enantiomers via β-CD additive is as efficient as using a dedicated 25-cm-long chiral Pirkle-type column, working under normal phase HPLC or supercritical fluid chromatography conditions [69, 70].

**Fig. 1:**
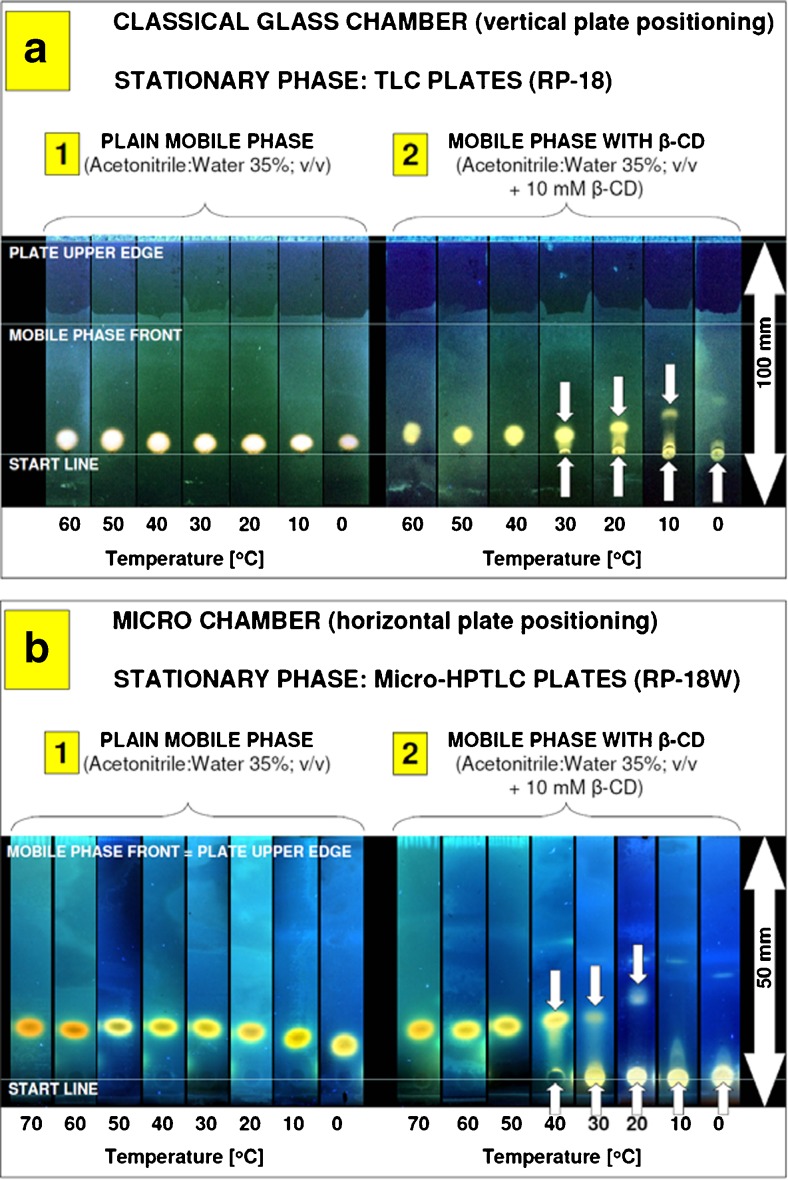
Planar chromatographic behavior of 1-acenaphthenol (racemic mixture) at different temperatures using RP-18 F_254_S classical TLC plates (**a**; vertical development) and RP-18 WF_254_S HPTLC microplates (**b**; horizontal development), involving plain binary acetonitrile:water mobile phase (*1*) and modified with β-cyclodextrin additive at concentration of 10 mM (*2*). Detection: fluorescence (366 nm/Vis). *Small arrows* indicate the main spots separated

In the past, we documented that in particular cases, planar chromatography may provide a more efficient separation system than column chromatography with regards to separation efficiency and peak distribution of samples composed of low molecular mass and low-retarded analytes (like estrogens) [71]. For that reason and taking into account successful HPLC enantioseparation of 1-acenaphthenol by β-CD additive at subambient temperatures, we conducted chromatographic studies under planar chromatography conditions (Fig. 1).

First approach was carried out using classical vertical glass chamber placed in water bath (working as temperature controlling device), and TLC plates coated with RP-18 stationary phase. Similarly to the HPLC system studied formerly, TLC mobile phase was composed of acetonitrile in water binary mixture (35%; *v*/*v*). By analogy to column chromatography, under such conditions high elution of macrocyclic additive can be expected (CD additive front should be more than *R*_*F*_ > 0.5 or close to the mobile phase front, preferably), and therefore, at subambient temperatures the interacting analytes should migrate close to the cyclodextrin additive front [39, 43]. As can be seen from planar chromatograms presented in Fig. 1(a1)**,** under plain, achiral binary mobile phase conditions, there is almost no temperature effect on 1-acenaphthenol retention. This compound is slightly eluted from the start line (*R*_*F*_ ranging from 0.06 to 0.1). After mobile phase modification by β-CD additive (10 mM; Fig. 1(a2)), significant effect of macrocyclic additive has been observed. Starting from temperature 30 °C and below, the presence of analyte fraction remaining on the start line can be observed. At ambient temperature, some quantity of 1-acenaphthenol is beginning to elute along the plate, driven by interaction with β-CD additive. This observation is in agreement with our basic retention concept (strong interaction of low retarded cyclodextrin molecules with analyte), which is occurring in mobile phase. Surprisingly, at subambient temperature (10 °C), the fraction of analyte that remains at start time is dominating. At lowest temperature investigated (0 °C), almost 100% of 1-acenaphthenol quantity remains at the start line. Similar phenomenon was not observed under HPLC conditions: no massive decrease of the peaks area for separated 1-acenaphthenol enantiomers at subambient temperatures was observed.

It should be noted that contrary to column chromatography, a non-forced flow planar chromatography, due to the presence of gas phase, is strongly affected by several additional parameters. They might be critical for separation results like chamber geometry and volume, plate positioning: horizontal/vertical, development mode: saturated/non-saturated, stationary phase type: classical RP-18, or wettable with water RP-18 W (different density of octadecyl chains on the stationary phase surface). To eliminate potential instrumental problems, which may cause the phenomenon observed, planar chromatographic experiment was repeated using different reversed stationary phase (HPTLC RP-18 W; wettable with water octadecylsilane) and equipment conditions providing high stability of given temperature and horizontal positioning of the plate during chromatographic run (microchamber unit working within insulated, temperature-controlled metal oven) [50]. Obtained results have confirmed typical Van’t Hoff behavior of acenaphthenol for the plain mobile phase (Fig. 1(b1) and Table S1 in the Electronic Supplementary Material, ESM) and strong retention of 1-acenaphthenol at low temperature using cyclodextrin modified mobile phase (Fig. 1(b2)). There are some differences in retention range of 1-acenaphthenol on TLC and HPTLC plates but they can be associated with, e.g., different properties of stationary phases applied. Accordingly to the plates Certificates of Analysis, provided by Merck to each plates batch, the retention of testing marker (cholesterol) is different. Reported *hR*_*F*_ values are equal to 40 for RP-18 TLC plates and 58 for HPTLC RP-18 W plates (Table 1). Similarly, for plain binary mobile phase studied in our experiment, retention of acenaphthenol is stronger on TLC plates (Fig. 1(a1)) than on HPTLC plates (Fig. 1(b1)). As can be seen from the spots pattern presented in Fig. 1(b2), a strong adsorption of acenaphthenol on the start line was observed starting from 30 °C.

**Table 1:** Physicochemical and chromatographic parameters of TLC silica gel 60 RP-18 F_254_S and HPTLC silica gel 60 RP-18 WF_254_S glass plates listed in Certificates of Analysis provided by Merck to each plates batch

Parameter	Specification	Batch values
A: TLC layer (RP-18 F_254_S)		
Specific surface area (according to BET; 5-Pt. measurement) [m^2^ g^−1^]	480–540	511
Pore volume (N_2_ isotherm) [mL g^−1^]	0.74–0.84	0.78
d 50 (laser diffraction, size distribution) [μm]	9.5–11.5	11.4
Layer thickness [μm]	200–270	210
Deviation of layer thickness per plate	≤35	10
Chromatography test (cholesterol elution) [hR_F_]	31–43	40
B: HPTLC layer (RP-18 WF_254_S)		
Specific surface area (according to BET; 5-Pt. measurement) [m^2^ g^−1^]	480–540	521
Pore volume (N_2_ isotherm) [mL g^−1^]	0.74–0.84	0.8
d 50 (laser diffraction, size distribution) [μm]	5–7	6
Layer thickness [μm]	150–200	170
Deviation of layer thickness per plate	≤35	15
Chromatography test (cholesterol elution) [hR_F_]	54–66	58

Considering that both planar chromatography experiments were different in terms of experimental setup including chamber geometry, volume, plates positioning (vertical, horizontal), development modes (saturated, unsaturated), and stationary phase properties (classical RP-18 and wettable with water RP-18 W as well as TLC/HPTLC: particle size and layer thickness; Table 1), observed adsorption phenomenon using β-CD mobile phase additive can be considered as general for studied target analyte, chromatographed under reversed phase planar chromatography conditions.

**Fig. 2:**
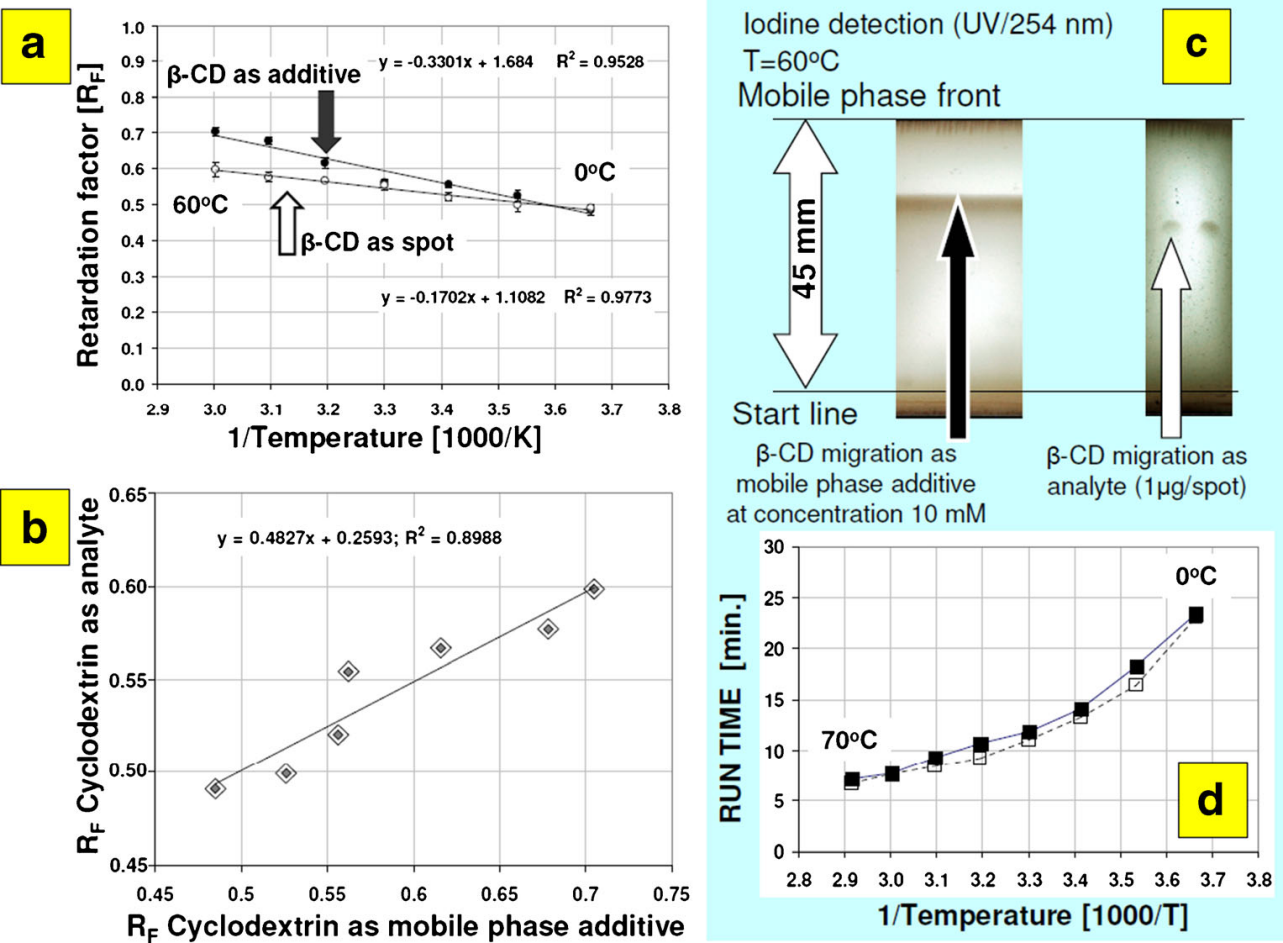
β-Cyclodextrin chromatographed as analyte (spot position) and mobile phase additive at concentration of 10 mM (β-CD front position) on HPTLC RP-18 WF_254_S plates at different temperatures using 35% (*v*/*v*) acetonitrile in water mobile phase: retention data (**a**), correlation between two retention modes (**b**), examples of micro-chromatograms (**c**), and run time for mobile phase migration at distance of 45 mm (**d**) measured for plain mobile phase (*empty squares*) and eluent modified with 10 mM β-CD (*black squares*)

To investigate this effect more closely, retention of β-cyclodextrin using binary mobile phase acetonitrile:water 35:65 (*v*/*v*) was studied. Accordingly to data presented in Fig. 2 and Table S2 (ESM), Van’t-Hoff retention behavior of this macrocycle on HPTLC RP-18 microplates within temperatures investigated were observed. It has been confirmed that under such experimental conditions, elution of β-CD exceed or is close to *R*_*F*_ = 0.5 (Fig. 2a). Therefore, this system provides the principal requirement of low retention of macrocyclic additive acting as complexation agent. It should be noted that using acetonitrile:water mixtures, elution of β-CD can be even more optimal (close to the mobile phase front) using different acetonitrile concentration ranging from 40 to 60% (*v*/*v*). Using such experimental setup, the *R*_*F*_ values exceeding 0.9 were observed for all native cyclodextrins (α, β, and γ) at temperature of 30 °C [38]. We also documented that migration of β-cyclodextrin additive on the plate can be simply evaluated using retention data of β-CD chromatographed as analyte (Fig. 2b, c). Under temperatures investigated, the run time involving 50 mm microplates (45 mm developing distance) varied between 7 to 25 min, despite mobile phase composition (with or without cyclodextrin additive; Fig. 2d). Obtained results have revealed that at subambient temperatures, TLC elution of cyclodextrin is at least *R*_*F*_ = 0.5, which provides enough space for elution of β-CD/1-acenaphthenol complex, similarly to column chromatographic conditions.

**Fig. 3:**
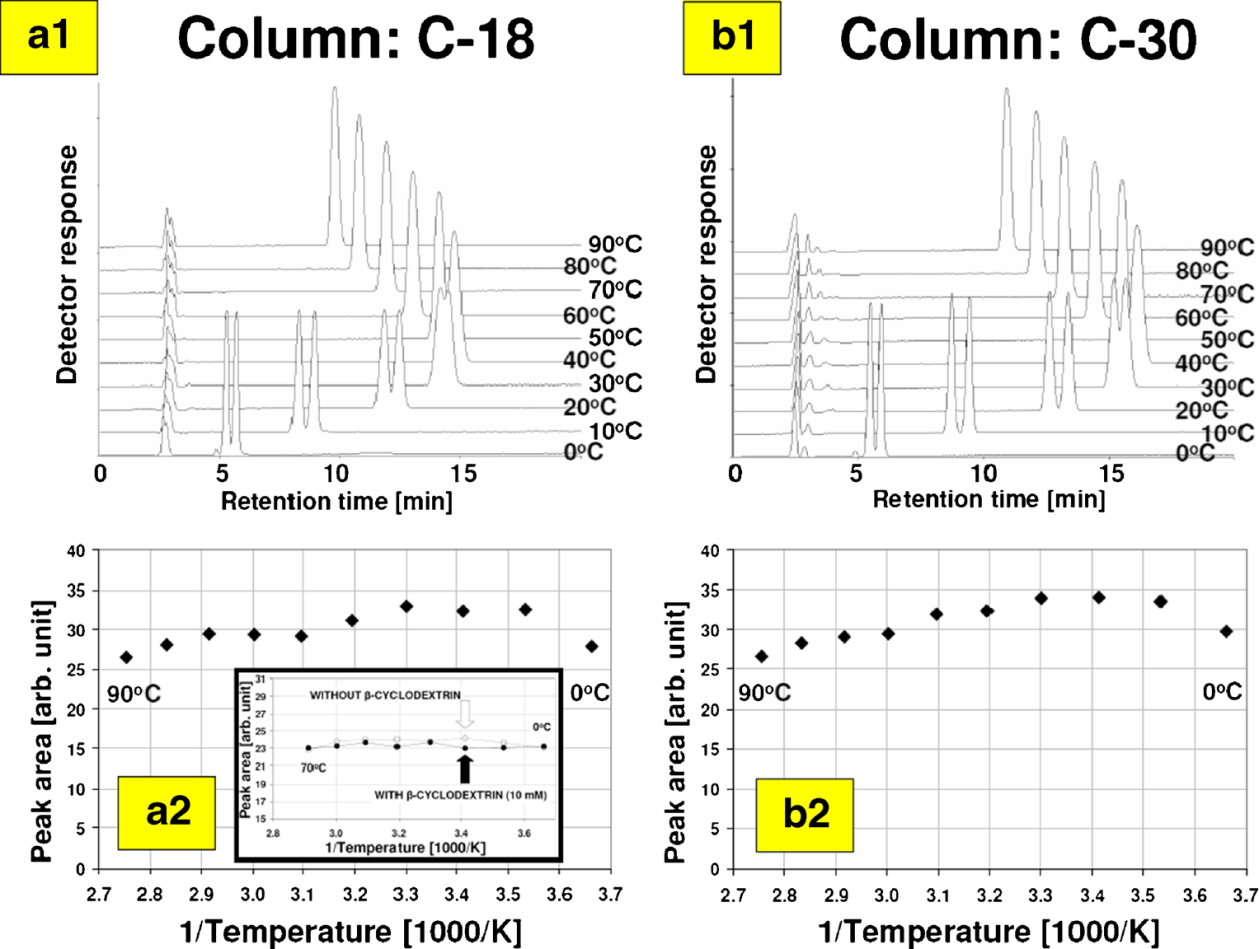
Separation of acenaphthenol enantiomers under HPLC conditions using β-CD additive to binary mobile phase composed of acetonitrile:water (35:65, *v*/*v*) using C-18 (**a1**) and C-30 (**b1**) stationary phases (both 15 cm long columns; mobile phase flow 0.5 mL/min) at different temperatures and corresponding peak integration results (**a2**, **b2**; peak areas for separated enantiomers were summarized). Graph inserted within plot **a2** refers to acenaphthenol peak area data obtained on Supelcosil LC-18 column (10 cm, flow 1 mL/min) without and with β-cyclodextrin additive (labeled as *empty circles* and *black dots*, respectively)

Due to registering the inconsistent retention results of 1-acenaphthenol using reversed-phase planar and column chromatographic systems (operating at subambient temperatures with mobile phase modified by β-CD additive), HPLC retention of 1-acenaphthenol was closely inspected. Moreover, using different stationary phases (C-18 and C-30), we checked if there is the peaks area decreasing phenomenon at subambient temperatures, indicating potential strong adsorption of the 1-acenaphthenol fraction on the column front (no elution from the column that is equivalent of strong retention on TLC plate). Data presented within Table S3 (ESM) show linear Van’t Hoff behavior of 1-acenaphthenol chromatographed under RP-HPLC conditions involving plain binary mobile phase acetonitrile:water 35:65 (*v*/*v*). Similar linear behavior of 1-acenaphthenol was observed using planar chromatography systems as described above. According to HPLC chromatograms presented within Fig. 3 (a1 and a2), increase of 1-acenaphthenol retention on both tested stationary phases (C-18 and C-30) was observed, indicating no or very week interaction with β-CD to mobile phase additive. In both HPLC systems, enantioseparation of 1-acenaphthenol isomers occurred at temperature close to 30 °C. At ambient and subambient temperature regions, baseline separation of 1-acenaphthenol enantiomers is observed, which is associated with fast peaks elution from the column (and deviation from Van’t Hoff plot), due to interaction with β-CD additive low retarded by stationary phase. This phenomenon was observed before and documented experimentally for various low-molecular mass chiral and achiral molecules (including polycyclic aromatic hydrocarbons, steroids, and bisphenols) chromatographed under similar conditions [9, 41–43]. As can be seen from plots a2 and b2 presented within Fig. 3, there is no massive loss in the peaks areas of 1-acenaphthenol at different temperatures using cyclodextrin-modified eluent. Observed variations of the peak areas can be associated with UV-Vis spectra changes of 1-acenaphthenol-CD complex at different temperatures due to the fact that UV detector cell of this HPLC system was not temperature controlled. In such case, temperature of mobile phase inside detector cell can be affected by temperature of mobile phase pumped from HPLC column (no heat stabilizer was placed between column thermostatic jacket and detector flow cell).

Effect of temperature on UV-Vis spectra was observed before for several molecules including dyes, alcohols, anti-inflammatory drugs, and polycyclic aromatic hydrocarbons, where both hypsochromic and bathochromic shifts as well as hyperchromic and hypochromic effects were reported [40, 43, 66, 67]. These phenomena are commonly used for calculation of binding constants of supramolecular complexes [3]. Noteworthy, if thermostated UV detector cell was applied, there is no effect on the peaks area for both modified and unmodified mobile phases as demonstrated for another HPLC machine equipped with temperature-controlled detector cell and temperature set at 30 °C (plot inserted within graph a2 in Fig. 3).

From practical point of view, any loss in the peak area may affect the accuracy and robustness of quantification protocol for components of interest, which are analyzed with help of cyclodextrin-modified liquid chromatographic system. Therefore, we strictly inspected all previously generated data, in term of this problem. Our investigation revealed that this problem does not exist for a number of various analytes and very well mobile phase soluble hydroxypropyl derivative of β-cyclodextrin (Fig. S1 in the ESM) [41]. In case of native β-CD additive, potential problem was observed for two steroids: progesterone and 20α-hydroxyprogesterone eluted for temperatures below 10 °C. It has been found that in temperature 0 °C, around 50% peak loss can be expected for these steroids. Based on this observation and considering that both analytes interact strongly with β-cyclodextrin (however, resulting complexes are characterized by relative strong retention—remain long time on the column), we hypothesized that solubility/precipitation kinetics of complexes may be responsible for the peaks loss in both column and planar chromatographic systems. Simply, strong interacting molecules with β-cyclodextrin may escape the column faster at low temperature but in planar chromatography, all analytes remain on the plate as long as the chromatographic run is finished (mobile phase must reach given distance). As we documented above, TLC chromatographic run is longer at low temperature than at high temperature despite of the mobile phase composition (with or without β-cyclodextrin). At low temperature (0 °C), overall development of TLC plate last more than 25 min while under HPLC conditions, 1-acenaphthenol remains on the column only 5 min, approximately. There is also a difference in acenaphthenol quantity injected into both systems. Particularly, 0.2 μg of analyte (20 μL loop and 10 μg mL^−1^ acenaphthenol solution) was injected into the analytical columns (4.6 mm ID) operating with flow rate ranging from 0.5 to 1.0 mL min^−1^, whereas TLC experiments were performed using 1 μg/spot acenaphthenol mass located within stationary phase volume of 2 μL, approximately (estimated total volume of mobile and 200-μm-thick stationary phase, corresponding to average HPTLC spot area = 11 mm^2^ for spot diameter = 3.8 mm). It should be noted that in both cases, investigated chromatographic systems were not overloaded (spot or peaks tailing were not observed) but initial local concentration of 1-acenaphthenol can be significantly lower on column than on plate.

**Fig. 4:**
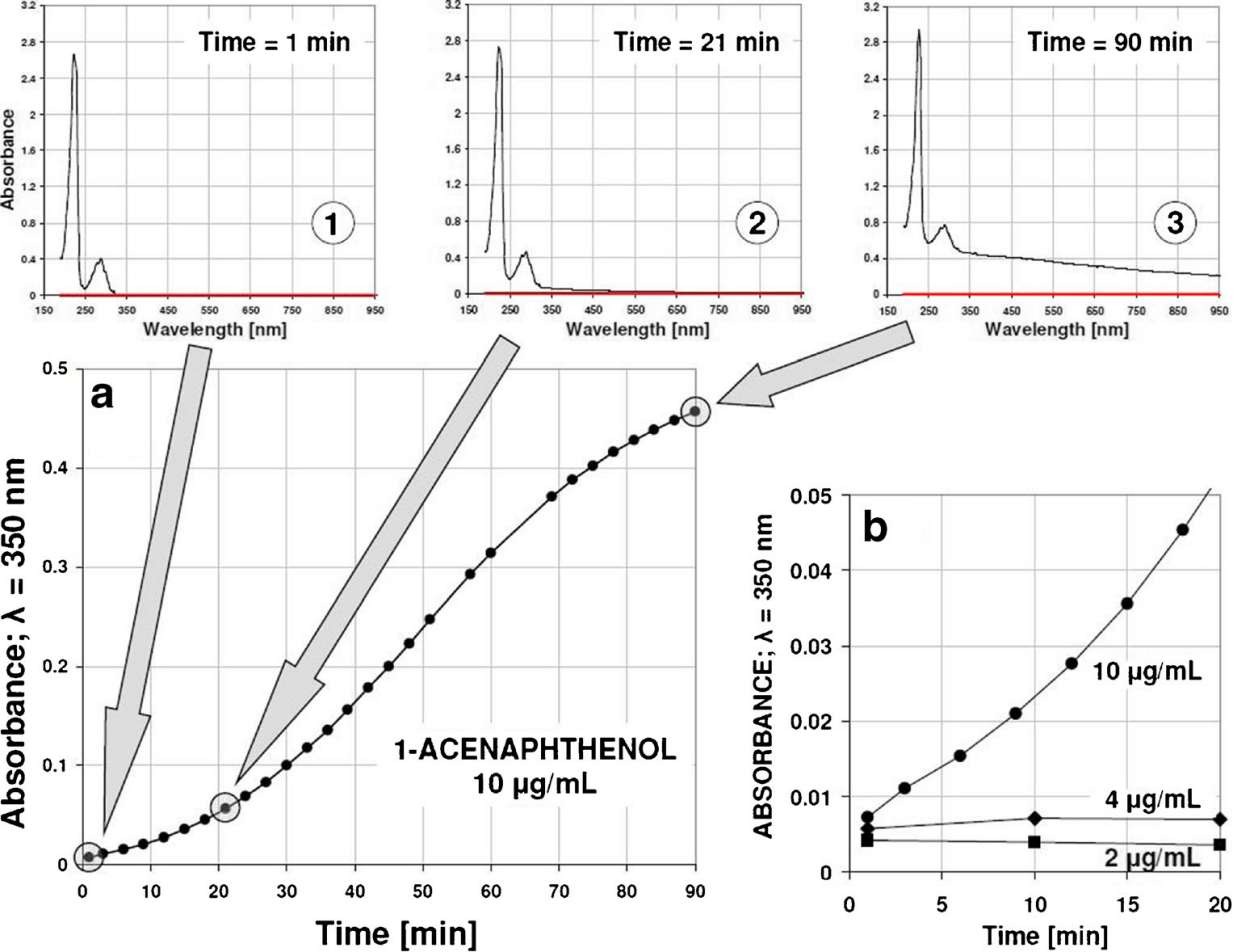
UV-Vis detection of crystallization phenomenon observed for 1-acenaphthenol and β-cyclodextrin complex in acetonitrile:water (35:65, *v*/*v*) liquid phase (measurement temperature: 20.0 ± 0.1 °C). **a** Background increase monitored at 350 nm for 1-acenaphthenol at concentration of 10 μg/mL and 10 mM β-CD (UV-Vis spectra presented at top were recorded for given crystallization times: 1, 21, and 90 min.). **b** Comparison of solid complex creation for different 1-acenaphthenol concentrations 2, 4, and 10 μg/mL using β-cyclodextrin at concentration of 10 mM

**Fig. 5:**
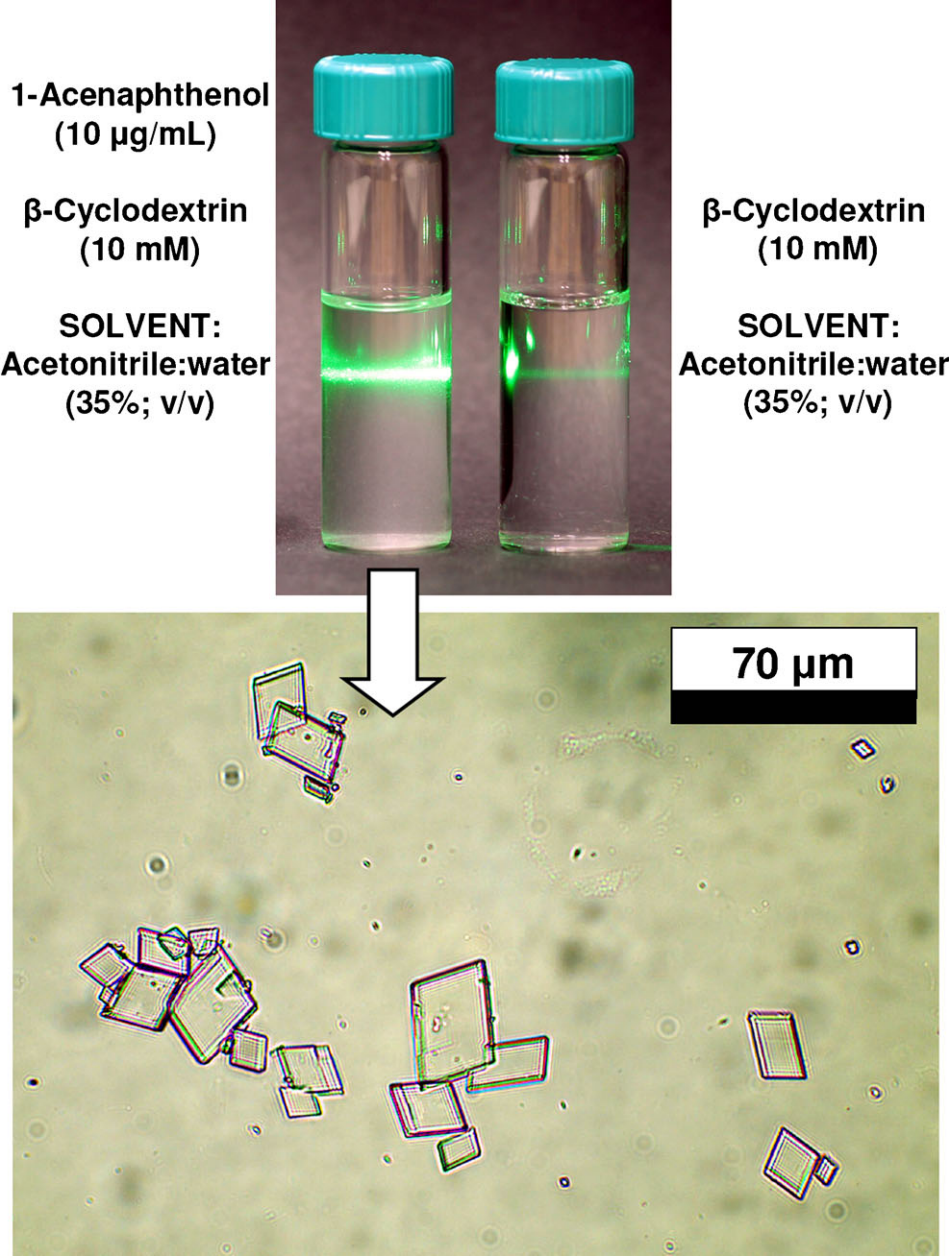
Visible light scattering (*green laser beam*; 532 nm; <10 mW) observed for solid particles of supramolecular complex generated from 1-acenaphthenol (10 μg/mL) and β-cyclodextrin (10 mM) mixture in acetonitrile:water (35:65, *v*/*v*), liquid phase, after 3 days at room temperature (22 ± 1 °C) conditions (*top*) and optical microscope view of precipitated crystals (*bottom*)

To confirm a hypothesis that solubility changes of β-CD/1-acenaphthenol complex may trigger strong retention in given chromatographic system, the acetonitrile:water solutions containing 1-acenaphthenol and β-cyclodextrin at concentration corresponding to chromatographic experiments (10 mM) were prepared and UV-Vis spectra registered under temperature-controlled conditions (20.0 ± 0.1 °C). UV-Vis spectrophotometric data presented in Fig. 4 clearly indicated that using relatively low concentration of 1-acenaphthenol (10 μg mL^−1^) in solution containing β-cyclodextrin, the solid complex can be formed and precipitate. This was detected by increase of baseline level within whole UV-Vis spectrum range (Fig. 4, spectrum No 3), driven by light scattering from the crystals formed. This effect is time-dependent as demonstrated on graph A in Fig. 4 **.** Moreover, it has been found that at a temperature of 20 °C, the investigated complex is soluble if 1-acenaphthenol concentration is less than 4 μg mL^−1^ (Fig. 4b). Light scattering from the solid supramolecular complex crystals can be also visualized using laser beam as can be seen from the upper picture in Fig. 5. Close inspection of centrifuged solid particles by optical microscopy (bottom picture in Fig. 5) confirms the presence of supramolecular complex crystals generated from 1-acenaphthenol and β-cyclodextrin mixture.

## Conclusions

1-Acenaphthenol enantiomers can be efficiently separated at ambient and subambient temperatures using reversed-phase HPLC systems and involving mobile phase modified with β-cyclodextrin additive (10 mM) and various C-18 and C-30 columns. Significant differences between planar and column chromatographic behavior of 1-acenaphthenol at ambient/subambient temperatures using mobile phase modified with β-cyclodextrin has been demonstrated based on various TLC and HPLC conditions.

We demonstrated that solubility changes of studied supramolecular complex and kinetics of solid complex precipitation as well as differences in total analysis time between TLC and HPLC separation may trigger strong retention of 1-acenaphthenol in planar chromatographic systems. In particular cases (long retention time of β-CD/host molecule complex), quantification of low-molecular analytes using HPLC mobile phase modified with native β-cyclodextrin may be affected by precipitation phenomenon of supramolecular complex created. This may occur at subambient temperatures close to 0 °C. Such disadvantage can be eliminated using, e.g., more soluble hydroxypropyl-β-cyclodextrin instead native β-CD additive.

Presented data have revealed that the solubility properties of supramolecular complexes with native β-cyclodextrin may be critical for modeling of chromatographic retention driven by host-guest interaction. Moreover, supramolecular complex precipitation combined with plates or bars solid phase extraction can be applied as very selective method for low-molecular mass components fractionation or separation involving micro-chromatography or microfluidic devices.

Solubility decrease of host-guest complexes with native β-cyclodextrin may be applied to design efficient water purification systems, which can be highly selective for given low-molecular mass micropollutants.

It has been demonstrated that contrary to column chromatography, where analytes must be eluted from the column to reach the detection place, classical non-forced flow planar chromatographic system can be very useful tools for examining the behavior of analytes that are strongly adsorbed by the stationary phase (retarded, chemisorbed, or precipitated within or close to the start line).

## Electronic supplementary material

ESM 1 (PDF 278 kb)
