# Supplementary material for: Unexpected differences between planar and column liquid chromatographic retention of 1-acenaphthenol enantiomers controlled by supramolecular interactions involving β-cyclodextrin at subambient temperatures
Source: Anal Bioanal Chem. 2017 Mar 24;409(14):3695–706. doi: 10.1007/s00216-017-0313-y (PMC5406416; doi:10.1007/s00216-017-0313-y)
Supplement: Supplementary file 1 — (PDF 278 kb) [file 216_2017_313_MOESM1_ESM.pdf]

Analytical and Bioanalytical Chemistry

Electronic Supplementary Material

Unexpected differences between planar and column liquid chromatographic retention of 1-acenaphthenol enantiomers controlled by supramolecular interactions involving β-cyclodextrin at subambient temperatures

Hatsuichi Ohta, Elżbieta Włodarczyk, Krzysztof Piaskowski, Aleksandra Kaleniecka, Lucyna Lewandowska, Michał J. Baran, Mariusz Wojnicz, Kiyokatsu Jinno, Yoshihiro Saito, Paweł K. Zarzycki

Table S1 Regression coefficients (*intercept* and *slope*) and Pearson correlation coefficient (*r*) of the regression equations R_F = *intercept* + *slope*(1000/*T*) and log*k* = R_M = *intercept* + *slope*(1000/*T*) for acenaphthenol chromatographed on HPTLC microplates (RP-18 $WF_{254}S$) using mobile phase composed of acetonitrile:water 35:65 (v/v)

**Equation type**	***Slope***	***Intercept***	***r***
R_F = *intercept* + *slope*(1000/*T*)	-0.095<br>(0.020)	0.506<br>(0.064)	0.8924
log*k* = R_M = *intercept* + *slope*(1000/*T*)	0.279<br>(0.060)	-0.29<br>(0.19)	0.8861

Temperature ranging from 0 to 70°C; number of samples = 4; the values in parentheses indicate the standard errors of coefficients at a 95% significance level.

Table S2 Regression coefficients (*intercept* and *slope*) and Pearson correlation coefficient (r) of the regression equations RF = *intercept* + *slope*(1000/T) and log*k* = *RM* = *intercept* + *slope*(1000/T) for β-cyclodextrin chromatographed on HPTLC microplates (RP-18 WF254S) using mobile phase composed of acetonitrile:water, 35:65 (v/v)

A. β-Cyclodextrin as analyte (spot center)*

Equation type	*Slope*	*Intercept*	*r*
R_F = *intercept* + *slope*(1000/*T*)	-0.170<br>(0.012)	1.108<br>(0.039)	0.9886
log*k* = R_M = *intercept* + *slope*(1000/*T*)	0.299<br>(0.020)	-1.067<br>(0.068)	0.9886

B. β-Cyclodextrin as mobile phase additive (β-CD front)*

Equation type	*Slope*	*Intercept*	*r*
R_F = *intercept* + *slope*(1000/*T*)	-0.330<br>(0.033)	1.68<br>(0.11)	0.9761
log*k* = R_M = *intercept* + *slope*(1000/*T*)	0.604<br>(0.065)	-2.16<br>(0.22)	0.9722

* Temperature ranging from 0 to 60°C; number of samples = 3; the values in parentheses indicate the standard errors of coefficients at a 95% significance level.

Table S3 Regression coefficients (*intercept* and *slope*) and Pearson correlation coefficient (r) of the regression equation $\log k = R_M = intercept + slope(1000/T)$ for acenaphthenol chromatographed on octadecylsilane HPLC column (10 cm Supelcosil LC-18) using mobile phase composed of acetonitrile:water, 35:65 (v/v)

**Equation type**	***Slope***	***Intercept***	***r***
$\log k = R_M = intercept + slope(1000/T)$	0.432	-0.688	0.9974
	(0.013)	(0.042)	

Temperature ranging from 0 to 70°C; number of samples = 3; the values in parentheses indicate the standard errors of coefficients at a 95% significance level.

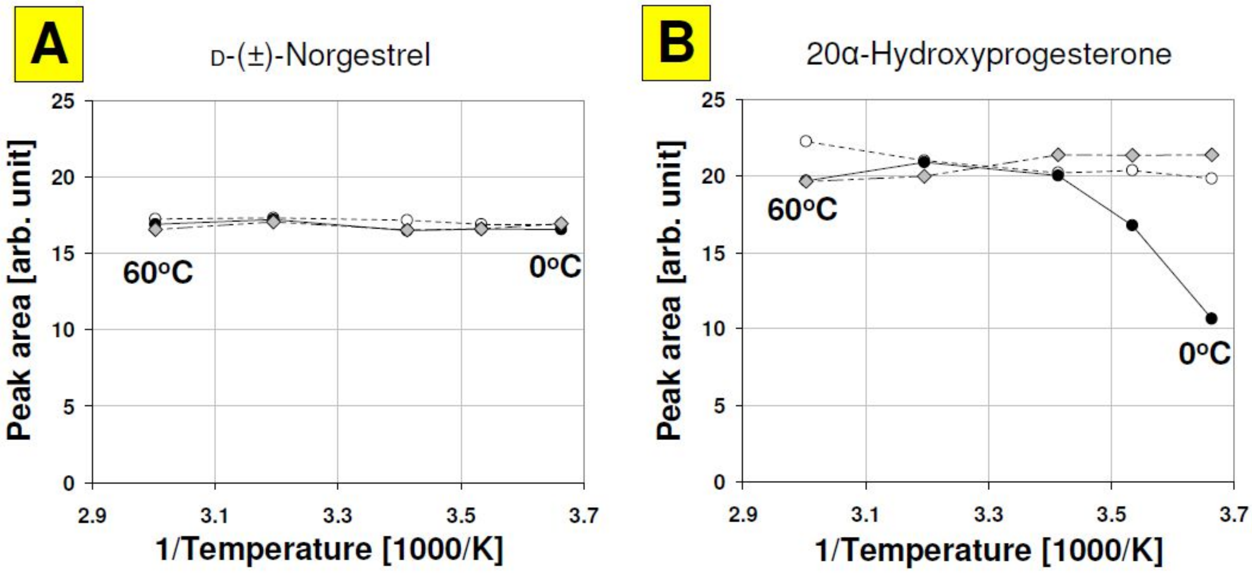

No effect on peak area:

Bisphenol A, 4-tert-Butylphenol, Cortisol, Cortisone, Diethylstilbesterol, 7,8-Dimethoxyflavone, Dimethyl phthalate, d-Equilenin, Equilin, Estetrol, 17α-Estradiol, 17β-Estradiol, Estriol, Estrone, Ethynylestradiol, 17α-Hydroxyprogesterone, Levonorgestrel, Medroxy-progesterone, Metyltestosterone, Norethindrone, Norgestrel, Testosterone, Tetrahydrocortisol, Tetrahydrocortisone, Toluene.

Peak area decreasing at subambient temperature using β-CD mobile phase additive:

Progesterone, 20α-Hydroxyprogesterone

Fig. S1 Temperature effect on peak areas of selected low-molecular mass compounds (mainly steroids) chromatographed on C-18 HPLC column using binary mobile phase composed of acetonitrile:water 35:65, v/v (white circles) and modified with β-cyclodextrin (black dots) as well as hydroxypropyl β-cyclodextrin (gray diamonds) additives at 10 mM concentration. A - no effect registered; B - peak area decreasing at low temperature using β-cyclodextrin as eluent additive. Steroids quantity injected: 20 μL of solution at concentration of 50 μg mL^{-1}; detection: UV 240 nm
